# Performance Differences of a Touch-Based Serial Reaction Time Task in Healthy Older Participants and Older Participants With Cognitive Impairment on a Tablet: Experimental Study

**DOI:** 10.2196/48265

**Published:** 2024-03-21

**Authors:** Christian Mychajliw, Heiko Holz, Nathalie Minuth, Kristina Dawidowsky, Gerhard Wilhelm Eschweiler, Florian Gerhard Metzger, Franz Wortha

**Affiliations:** 1 Geriatric Center University Hospital for Psychiatry and Psychotherapy University of Tübingen Tübingen Germany; 2 TuCAN Tübingen Cognitive Assessment for Neuropsychiatric Disorders Tübingen Germany; 3 Institute of Computer Science Ludwigsburg University of Education Ludwigsburg Germany; 4 LEAD Graduate School & Research Network University of Tübingen Tübingen Germany; 5 Vitos Hospital for Psychiatry and Psychotherapy Haina Klinik für Psychiatrie und Psychotherapie Vitos Haina gGmbH Haina Germany; 6 Centre for Early Mathematics Learning School of Science Loughborough University Loughborough United Kingdom

**Keywords:** serial reaction time task, SRTT, implicit learning, mobile digital assessments, cognitive impairment, neurodegeneration, tablet-based testing, mild cognitive impairment, MCI, dementia, Alzheimer, neuropsychology, aging, older individuals

## Abstract

**Background:**

Digital neuropsychological tools for diagnosing neurodegenerative diseases in the older population are becoming more relevant and widely adopted because of their diagnostic capabilities. In this context, explicit memory is mainly examined. The assessment of implicit memory occurs to a lesser extent. A common measure for this assessment is the serial reaction time task (SRTT).

**Objective:**

This study aims to develop and empirically test a digital tablet–based SRTT in older participants with cognitive impairment (CoI) and healthy control (HC) participants. On the basis of the parameters of response accuracy, reaction time, and learning curve, we measure implicit learning and compare the HC and CoI groups.

**Methods:**

A total of 45 individuals (n=27, 60% HCs and n=18, 40% participants with CoI—diagnosed by an interdisciplinary team) completed a tablet-based SRTT. They were presented with 4 blocks of stimuli in sequence and a fifth block that consisted of stimuli appearing in random order. Statistical and machine learning modeling approaches were used to investigate how healthy individuals and individuals with CoI differed in their task performance and implicit learning.

**Results:**

Linear mixed-effects models showed that individuals with CoI had significantly higher error rates (b=−3.64, SE 0.86; *z*=−4.25; *P*<.001); higher reaction times (*F*_1,41_=22.32; *P*<.001); and lower implicit learning, measured via the response increase between sequence blocks and the random block (β=−0.34; SE 0.12; *t*=−2.81; *P*=.007). Furthermore, machine learning models based on these findings were able to reliably and accurately predict whether an individual was in the HC or CoI group, with an average prediction accuracy of 77.13% (95% CI 74.67%-81.33%).

**Conclusions:**

Our results showed that the HC and CoI groups differed substantially in their performance in the SRTT. This highlights the promising potential of implicit learning paradigms in the detection of CoI. The short testing paradigm based on these results is easy to use in clinical practice.

## Introduction

### Memory, Neurodegeneration, and Aging

#### Overview

In an aging society, the number of individuals with neurodegenerative diseases is increasing. Alzheimer disease (AD) and Parkinson disease rank among the most prevalent neurodegenerative disorders. One of the most apparent cognitive symptoms of neurodegenerative diseases is a change in memory impairment, which can affect different cognitive and memory functions in different ways.

Memory functions differ in concepts and models [1-5]. When comparing different memory models, memory can be categorized into specific subsystems: intentional learning leads to explicit memory, and unintentional and incidental learning creates implicit memory [3-6]. Most studies on neuropsychological changes in neurodegenerative diseases focus on explicit memory, whereas studies examining the decline of implicit memory remain scarce [7-13]. Accordingly, many assessments for explicit memory are available, but only a few assessments are available for implicit memory, and most assessments are paper based. Paper-based examination of implicit memory is effortful and limited in its possibilities and test quality, although there are some digital tools available [8,14]. Still, solely examining explicit memory seems to be insufficient, as neurodegenerative disorders such as AD show changes in different cognitive domains, not just explicit memory but also implicit memory [14-19], among others. Thus, implicit memory paradigms are promising tools in addition to common explicit memory tasks in early diagnostics to assess memory dysfunctions more precisely and to determine the different causes of neuropsychological dysfunction. Reasons for reduced performance in implicit memory tasks can be independent of the reasons for deficits in regular tasks such as word lists used for explicit learning.

We introduce a digital tablet–based version of the serial reaction time task (SRTT) aimed at assessing implicit memory. We then use data gathered by this tool to develop machine learning (ML) models for predicting cognitive impairment without relying on diagnoses from comprehensive assessments such as the Montreal Cognitive Assessment or the Consortium to Establish a Registry for Alzheimer’s Disease. On the basis of computer-based studies, we developed an app for clinical application, aiming to provide results comparable with previous findings on the SRTT. This study examines a mobile touch–based SRTT on a tablet in healthy older participants and older participants with cognitive impairment (CoI).

#### Implicit Learning

Researchers use various terms for implicit memory, referring to different concepts and processes inconsistently. Sometimes, these terms can refer to subtypes of a form of memory [8,14]. Although *implicit learning* is an umbrella term for the absence of awareness and intention, which means “a collection of abilities that are expressed through performance without requiring conscious memory content” [2], *statistical learning* “refers to the ability to detect and learn regularities in the environment” [13]. *Sequence learning* “describes the ability to incidentally acquire knowledge of sequences of events and actions” [20]. *Motor skill learning* “refers to the increasing spatial and temporal accuracy of movements with practice” [21]. *Procedural learning* is used as a synonym for sequence learning, referring to the learning of sensory-motor skills [10,13,22,23]. As we are focusing on a rather application-oriented approach, we further refer to the more general concept of implicit learning.

#### SRTT Paradigm

**Figure 1:**
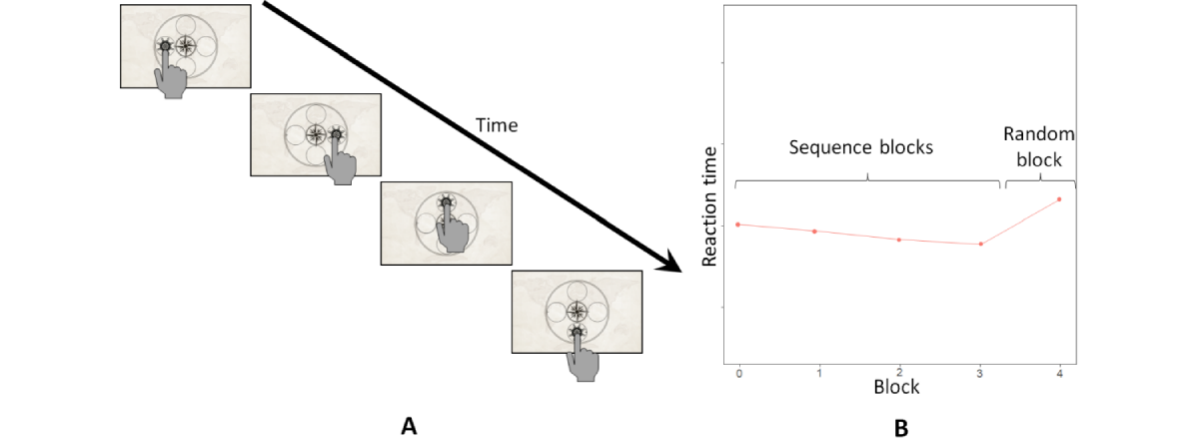
Illustration of the serial reaction task paradigm used in this study and the expected reaction times for healthy participants. (A) Touch-based variant used in the study. The participants’ task was to respond with their finger to the target stimulus in the form of a ship’s wheel on the tablet. (B) Expected reaction times for healthy participants. After the sequence is repeated in the first 4 blocks, leading to a decrease in participants’ reaction time, a random sequence occurs in block 5, resulting in a significant increase in participants’ reaction time.

Different tasks have been used to assess implicit learning [8,14]. Among others, the SRTT was established as a widely used assessment. In the original version of the SRTT [24], participants react to stimuli presented in blocks with repeated sequences. After several blocks of repeated sequences (“sequence blocks”), a block with random sequences is presented (“random block”). Although reaction times usually improve throughout the blocks of repeated sequences, they decelerate in the blocks with a random sequence [16,24]. Implicit learning is assumed when there is a decrease in reaction time in blocks of repeated sequences and an increase in reaction time in the random block. Figure 1 shows an illustration based on the example of this study.

#### SRTT Variants

There are many variations in the SRTT paradigm and ambiguous findings on the effects and results of different clinical syndromes. Variations in paradigms can significantly influence the patterns of results observed. Different versions of the SRTT may vary in factors such as the quantity and length of stimuli; number of trials and blocks; the arrangement, structure, and display of stimuli on screen; the method of response; and even the medium used. Stimuli are presented on a computer screen in most studies [14,25]. Participants are asked to indicate the position of the stimuli using buttons on a keyboard or button box. Very few studies used a touchscreen- or tablet-based presentation of the SRTT [9,26-31]. Thus, researchers have many degrees of freedom in adjusting the paradigms of the SRTT, especially when implementing a touch-based version for older participants. In this setup, the participants use their fingers to indicate the position of the stimuli directly on the touchscreen. The most appropriate specifications may vary based on the research question (RQ) and the sample under investigation.

As described by Hong et al [16], an alternating design, that is, a design with an alternating sequence and random blocks [32-34], has advantages in distinguishing between motor and cognitive learning, but progressions in sequence tasks cannot be analyzed. Moreover, alternating sequences lead to a longer overall assessment time. In contrast to laboratory studies, design decisions are limited in a clinical approach. An SRTT version suitable for clinical use with older participants should be as short as possible to meet their stamina and motivation, especially in the case of CoI. Although some researchers suggest the superiority of alternating SRTT variants because of their capacity to discriminate between sequence-specific and general skill learning [35], we decided to use the SRTT in a tablet-based version as a short SRTT version that only needs 5 blocks and thus is much shorter and more usable in clinical contexts.

#### Possible Distinguishing Features

The patient and control groups differ for various outcome measures. First, *reaction times* can differ between groups in general, meaning that healthy controls (HC) are faster than patient subgroups. Second, *learning curves* can vary between groups, meaning that HC participants should learn sequences faster. Third, the *response increase* between the groups may deviate. That is, the contrast in reaction times between sequence and random blocks becomes more pronounced after extensive learning of sequences, rather than just motor skill leaning or increased familiarity with the task. Finally, the *number of correct responses*, that is, the *response accuracy* (and vice versa *error rates*), is expected to differ between groups. That is, the CoI group should show more false responses than the HC group. In addition, when we combine these variables as features in a statistical model, we may discover findings not only about implicit memory but also about parameters such as limitations in task comprehension or altered reaction times, which serve as additional diagnostic information.

#### SRTT Findings

The SRTT and similar tasks to assess implicit learning have been used in numerous studies in different fields [25], reporting different variables, outcome measures, and results. Varying patterns of results can be explained by different design variations (eg, [32,33]) and experimental requirements and conditions [8,14]. In healthy adults, differences in response increase were found consistently between sequence and random blocks. There was a slight tendency of age-related deterioration in performance, learning, accuracy, and reaction times with higher age [20,36,37]. Worse performance can be observed in patients with strokes [38]. Numerous studies show a deterioration of performance in neurodegenerative disorders affecting the basal ganglia and the thalamus, such as Parkinson disease [25,39,40], and other neurodegenerative and neuropsychiatric diseases, such as Huntington disease [41-43] and Korsakoff syndrome [24].

Some SRTT studies were conducted with patients with AD as the clinical sample, whereas others were conducted with patients with mild cognitive impairment (MCI; for reviews, refer to the studies by de Wit et al [8] and van Halteren-van Tilborg et al [14]). Overall, there are mixed findings on the association between SRTT performance and cognitive impairment (such as AD, which can be a later stage of MCI). Comparing various studies poses a challenge because of differences in sample characteristics, such as the varying degrees of impairment severity (eg, MCI vs AD at different levels of severity), and the diverse inclusion criteria used for clinical samples (eg, specific diagnostic criteria vs various screening scores) [8]. In addition, inconsistent methodological approaches, including variations in the quantity and duration of stimuli, sequences, and blocks, further complicate comparisons between studies. Some studies revealed no significant differences in learning concerning response increase for participants with MCI compared with HC participants. That is, participants with MCI and HC participants showed similar response increases between sequence and random blocks [9,16,44,45], whereas other studies found differences [15,46]. Participants with AD, however, show less response increase than HC participants in some studies [47-49] and a comparable response increase in others [50-52].

Compared with learning curves, that is, the reduction in reaction time over sequence blocks, patients with MCI seem to have similar curves as HCs in most studies [9,15,16,44,45], whereas some studies found differences [46,48]. Patients with AD show more deficits than HCs, as indicated by a flatter learning curve in some studies [49,53], but this was not clearly evident in most studies [47,50-52].

When comparing accuracy or error rate, in some cases, no differences are found between participants with AD [41,51,52] and MCI [44,46]. In other cases, participants with AD [47,48,50] and MCI [16,45] differ from the reference groups.

In most cases, patients with MCI [16,46] and AD [47,50-53] had slower overall reaction time [54]. These differences can be explained by motoric demands, the experimental design of the tasks, and differences in sample selection. In addition, a large number of dementia diseases may be mixed pictures of different subtypes of dementia [55,56]. This phenomenon may further contribute to the divergent findings. In a review and meta-analysis, de Wit et al [8] discuss the difficulty of participants with AD in understanding and remembering the test instructions of SRTT paradigms. Most studies found differences in response accuracy and reaction times (with participants with CoI being slower than HC participants). These differences suggest that the understanding and execution of the task play a significant role in classifying differences between healthy individuals and individuals with CoI.

### This Study

We developed a mobile SRTT version suitable for testing implicit memory in a clinical routine (in contrast to an extensive laboratory assessment). Working with older patients in everyday clinical routines, we recognized the need for a short, understandable, and highly accepted digital assessment that medical professionals can use in point-of-care or bedside tests without requiring additional technical equipment. We also expect a short and tablet-based variant to address the difficulties in task understanding and remembering task instructions among patients with CoI reported by de Wit et al [8]. Using a tablet in neuropsychological testing has benefits in terms of the availability of new data sources and its applicability outside the laboratory [57,58]. Furthermore, digitalized testing enables tasks and measures that are impossible in pen-and-paper testing [57]. Using a touchscreen is a considerable relief for older patients, and particularly patients with CoI, compared with using a keyboard [59-62]. Furthermore, motivational effects must be considered when designing tasks for older participants [57,63]. Not only the design of the task but also the task parameters are essential: the length of the sequence and the frequency of repetitions should capture implicit learning but should not be unnecessarily prolonged. Using ML approaches that combine various parameters, we may predict participants’ cognitive status more accurately with less data than a traditional approach, which accommodates shorter assessments. Combining the SRTT with ML represents a more recent development in this research domain [9,10,16].

ML predictions based on these parameters can potentially lead to accurate predictions using fewer repetitions, which facilitate shorter assessments that are mandatory for acceptance in clinical outpatient and inpatient practice.

### Research Questions

This study focuses on the following RQs, which are centered on the question of whether the findings for the computer-based version of the SRTT can be replicated and transferred to the tablet-based version of the SRTT used in this study.

We investigated the following RQs:

1. Do participants with CoI and HC participants differ significantly in response accuracy?
  We expect participants with CoI to make more errors than HC participants.
2. Do HC participants and participants with CoI differ in their average reaction times during the learning phase?
  We expect that participants with CoI are systematically slower than HC participants.
3. Do participants with CoI and HC participants differ in implicit learning? That is, is the response increase in the random block compared with the learning curve significantly lower for participants with CoI?
  We expect that participants with CoI show less implicit learning than HC.
4. Do participants with CoI show a different learning curve during the learning phase than HC participants?
  We expect HC participants to show a steeper learning curve than participants with CoI, which should show a flatter learning curve.
5. Can we reliably predict participants’ groups using an ML prediction model?
  We expect to classify participants with an accuracy comparable with that of Hong et al [16]. That is, we expect the 80.9% found by Hong et al [16] to be within our 95 % CI of prediction accuracy.

## Methods

### Participants

We recruited and tested 49 older participants at the Geriatric Center at the University Clinic for Psychiatry and Psychotherapy in Tübingen, Germany. A total of 2 participants discontinued the experiment. One participant had to be excluded owing to a low response accuracy of 40%, which indicates a failure to understand and complete the task appropriately. We later identified 1 participant with significantly prolonged reaction times as an outlier and had to exclude this participant. Within the scope of this project, we also collected data from 11 participants with depression, which we excluded from the analysis of this study because of the focus on neurodegeneration. The remaining 45 participants (26 female individuals), aged between 52 and 87 (mean 68.4, SD 9.82) years, consisted of 27 HC participants and 18 participants with CoI. A list of inclusion and exclusion criteria is presented in Textbox 1.

We based the allocation of groups on experienced physician examinations, confirmed by an interdisciplinary team (physicians, psychologists, specialized therapists, and nurses), as most participants were known to us as patients of our (day) hospital and their caregivers or relatives. We also recorded the participants’ educational level. Subsequently, we converted the educational levels into corresponding years representing the time typically taken to achieve them. The descriptive statistics of the demographics of the sample are provided in Table 1.

**Inclusion and exclusion criteria.**

**Inclusion criteria**

- Adults aged ≥50 years
- Diagnosis of cognitive impairment (for patient group), confirmed by an interdisciplinary team
- Understanding and agreement of informed consent
- Participation on a voluntary basis

**Exclusion criteria**

- Unable to perform or a lack of understanding of the task requirements
- Visual impairment
- Refusal or inability to give informed consent
- Acute delirious or psychotic episode
- Acute medical or physical conditions

**Table 1:** Demographic data of the 2 groups (HC^a^ and CoI^b^; N=45).

Characteristics		HC (n=27)	CoI (n=18)	Total (N=45)	*P* value	
**Age (y)**						.002^c^
	Mean (SD)	64.89 (9.66)	73.67 (7.62)	68.40 (9.82)		
	Range	52-85	55-87	52-87		
**Education (y)**						.52^c^
	Mean (SD)	11.74 (3.21)	11.06 (3.80)	11.47 (3.43)		
	Range	8-17	8-19	8-19		
**Sex, n (%)**						.39^d^
	Female	17 (63)	9 (50)	26 (58)		
	Male	10 (37)	9 (50)	19 (42)		

^a^HC: healthy controls.

^b^CoI: participants with cognitive impairment.

^c^Linear model ANOVA.

^d^Pearson chi-square test.

### Materials

#### Overview

**Figure 2:**
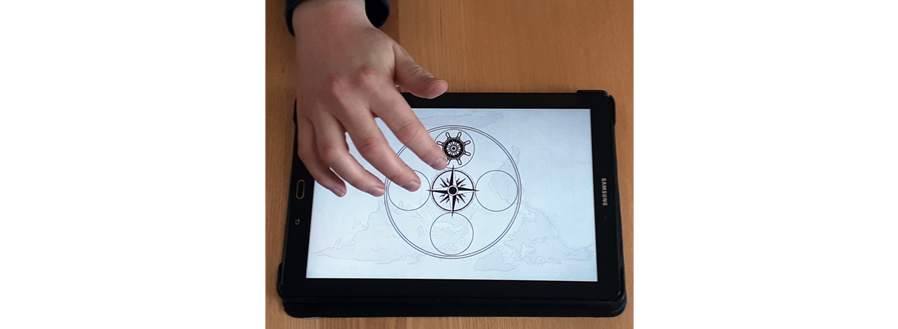
View of the setup and task in the app used for the study. Participants were allowed to rest their hands on the table.

We used a tablet-based variant of the SRTT (described in the subsequent sections) designed for this study to meet the needs of older participants. The experiments were performed on a “Samsung Galaxy Tab A (2016) with S Pen” tablet (model SM-P580, Samsung Electronics) with a screen size of 10.1 inches, running on Android 7.0. Tablets were positioned on the table horizontally and planar in front of the participants (Figure 2). Thus, the participants were able to rest their elbows on the table. The participants’ task was to repeatedly respond as quickly as possible to the target stimulus, whose position changed, with their fingers.

#### SRTT Design

**Figure 3:**
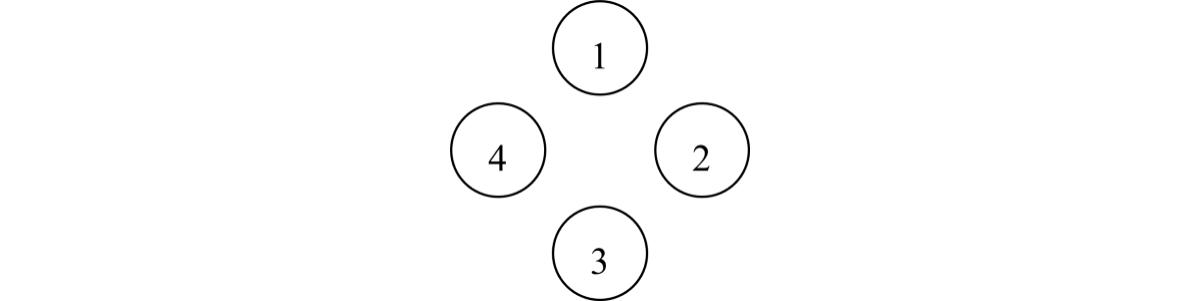
Circular order of the serial reaction time task.

The experimental design of the SRTT used in this study is based on the study by Lum et al [64], as they used a shorter version with fewer trials than previous studies, which was necessary in working with older participants. Thus, following a 10-trial practice phase, the SRTT used in this study consisted of 5 blocks with 60 trials each. Blocks 1 to 4 are sequence blocks and contain the 10-item sequence, repeated 6 times per block. The repeated sequence 4-2-3-1-3-2-4-3-2-1 is based on the original design of Nissen and Bullemer [24] and was also used by Lum et al [64] and Lum and Kidd [65]. In the circular order we used, the top position (north) corresponds to 1, 2 corresponds to the right-hand position (east), 3 corresponds to the lowest position (south), and 4 corresponds to the left position (west; Figure 3).

When we developed the tablet-based variant, a circular order of stimuli [9,10,64,66-69] was chosen to ensure comparable spatial distances between stimuli on the screen (Figure 3). A horizontal arrangement of stimuli would lead to unequal distances between stimuli. A touchscreen-based version of the SRTT was used in only a few studies in general [26,29,31,70], specifically in samples consisting of older individuals [27]. To the best of our knowledge, apart from the study by Dominey et al [27], no study with older participants that exclusively used touchscreen versions of the SRTT on a larger sample was published. The motor skills required for responding on a tablet surface differ from those needed for pressing buttons on a response panel [29,31].

In *block 5,* based on the study by Lum et al [64], the stimulus appears in a pseudorandomized order. This order is based on 2 conditions: first, each stimulus appears as often as in the antecedent (learning) sequence blocks, and second, the probability of appearing at 1 of the 4 positions after its antecedent stimulus is the same as in the learning sequence. We precomputed 1 pseudorandomized sequence (Multimedia Appendix 1) and used the same pseudorandomized sequence for each participant. We did not inform participants about the given configuration. Using the difference between sequential and pseudorandomized trials yields a measure of skill acquisition from the SRTT that is specific and sensitive, as measuring implicit learning by only comparing improved reaction times in sequenced blocks is confounded by visuomotor association [71]. Multiple parameters captured through the app were used for statistical modeling to assess their predictive value, via the approach of Hong et al [16] using random forest classification.

After the stimulus appears at position 1, the probability that it will appear at either position 3 or position 4 is 50%, respectively. After appearing at position 2, it is equally likely that the stimulus appears next at positions 1, 2, or 4, corresponding to 33% for each position. After appearing at position 3, the probability for the stimulus to appear next is 33.33% for position 1 and 66.66% for position 2. After appearing at position 4, the probability for the stimulus to appear next at position 2 or position 3 is 50%, respectively. After a practice trial, the participants were advised to react to the stimulus, changing their position as quickly as possible throughout the blocks. The app recorded the reaction times and the correctness of the reactions.

#### Task Implementation

**Figure 4:**
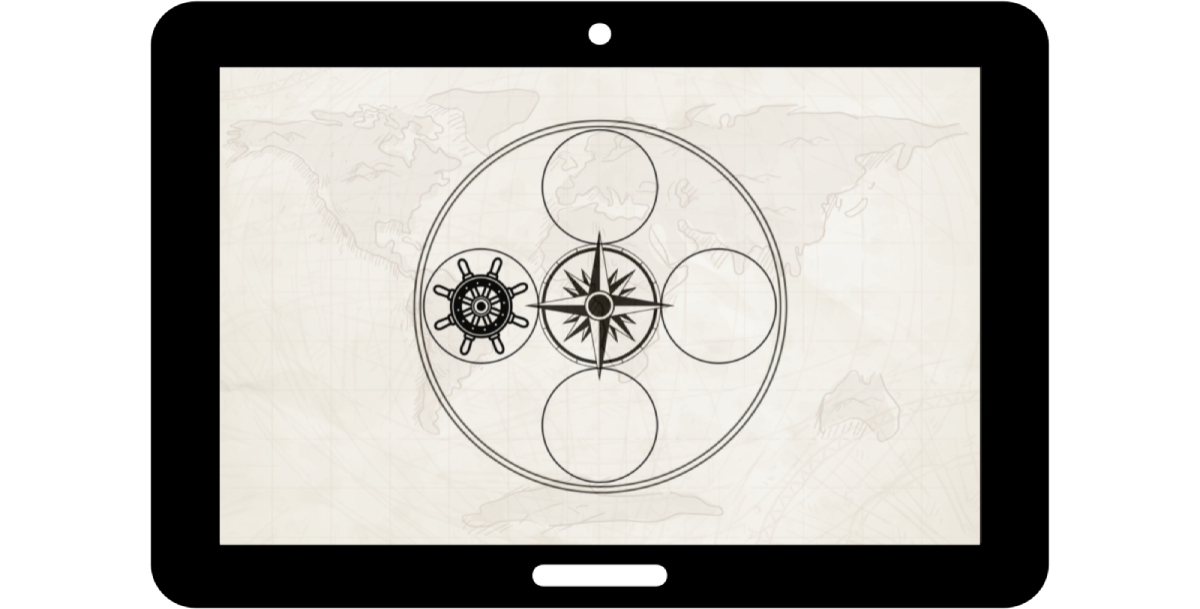
The compass design of the serial reaction time task developed to meet the needs of older participants.

We developed the mobile touch–based SRTT in Unity 3D, version 2019.1.0f2 [72] as part of the TuCAN (Tübingen Cognitive Assessment for Neuropsychiatric Disorders) Project, which develops a tablet-based test battery app. In a first pilot study with university students, we showed that different user interface designs on the tablet are comparable and that no effects are attributable to the design. Moreover, we examined the usability and preferences of different designs with older participants in a second pilot study. The usability study is substantial for older participants to accommodate for possible low computer and tablet literacy and to ensure that an app is developed according to the needs of older participants [60,73,74]. In the preceding user tests and pilot studies described in this section, in which different designs were compared, we identified a circular compass design as the preferred design version for older participants (Figure 4). In this design, a compass dial is placed in the center of the screen and is surrounded by 4 circles. In the background, an ancient-looking map is depicted. A ship’s wheel, as the target stimulus, changes the positions between the 4 circles.

### Statistical Analysis

#### Overview

We performed analyses on the full sample of 45 participants. In addition, because of significant differences in age between the groups, all statistical analyses were rerun using an age-matched subsample of 36 participants (18 HCs and 18 participants with CoI). The pattern of the results remained identical, underlining the robustness of the findings to age differences. Therefore, we only report the results from the full sample in this study. The results of the matched samples are reported in the web supplement [75]. Similarly, the assumptions for all statistical models were checked. In case of assumption violation, we reran the analyses with robust models to ensure that the pattern of results remained identical. For the sake of readability, the results of the robust models are only reported in the web supplement [75].

#### Analyses Software

We conducted statistical analyses for RQs 1 to 4 using R software (R version 4.3.2, R Foundation for Statistical Computing) [76].

We used the *lme4* package (version 1.1-35) to fit (generalized) linear mixed-effect models [77]. The df and *P* values were calculated with the *lmerTest* package (version 3.1-3) [78] using Satterthwaite approximation for the denominator df. We calculated the CIs for logistic regressions with the *broom.mixed* package (version 0.2.9.4) [79] using Wald approximation. We used the *ggplot2* package (version 3.4.4) [80] to create plots, the *stargazer* (version 5.2.3) [81], the *arsenal* (version 3.6.3) [82], and *tab_model* from the *sjplot* (version 2.8.15) [83,84] packages to create tables.

The criterion of statistical significance was set at Cronbach α=.05. The raw data and *R* scripts detailing all analyses can be accessed in the web supplement [75].

#### Data Cleaning

For all analyses except for the accuracy analysis (RQ 1), we removed trials within blocks according to the following criteria in the following order: (1) the first trial of each block, as these trials succeed the fixation cross; (2) erroneous trials; (3) trials following erroneous trials; (4) trials with reaction times <200 ms; and (5) trials with reaction times deviating >2.5 SDs from the mean within a block, within participants. In total, we removed 5.36% (723/13,500) of trials.

#### General Modeling Approach (RQ 1+RQ 3+RQ 4)

Mixed-effect models will be hierarchically constructed from a full model (containing all fixed and random effects, including interactions) with a maximum random effects structure to the model with the best fit according to the Bayesian information criterion by removing the most complex fixed effects first (ie, interaction terms). If the complexity of the random effect structure is not supported by the data (ie, convergence issues), the random effects structure is reduced, similar to the fixed effects structure, by removing the most complex terms first.

#### Accuracy (RQ 1)

To contrast the difference in accuracy between the 2 groups, we ran generalized linear mixed-effects models using the logistic link function in all trials. Accuracy was computed as the proportion of correct trials to the total number of trials per participant per block. The models included group, block, their interaction, and age as fixed effects and random intercepts for the participant.

#### Average Reaction Time (RQ 2)

Analyses of covariance (ANCOVAs) were performed on the cleaned data (ie, correct trials only) to contrast the difference in the average reaction time across the learning phase, that is, excluding the random block. The average reaction time was computed per participant as the average of the median reaction time of each block. The fixed effects were group, with age, sex, and years of education as covariates.

On the basis of recommendations for good scientific practice for reporting ANCOVAs [85], an ANOVA comparing the average reaction times between groups was also performed, showcasing the impact of the covariates on the results.

#### Implicit Learning (RQ 3) and Learning Curve (RQ 4)

Linear mixed effect (LME) spline models with the last sequence block as the knot were performed on participants’ median reaction times per block to contrast the influence of group, block, response increase, sex, age, education, and the interaction between block and group and response increase and group. The model included random slopes for block and response increase for the participants. The linear and quadratic effects of blocks were tested for their contribution to the model fit. To this end, orthogonal polynomials were computed to encode the linear and quadratic effects of time. The response increase between the expected reaction time in the random block, based on the estimated learning curve during the learning phase in the sequence blocks, and the measured reaction time in the random block was coded as follows: We used dummy coding for the response increase between the expected reaction time in the random block, based on the estimated learning curve during the learning phase in the sequence blocks and the measured reaction time in the random block. That is, the dummy variable for response increase is set to 0 for blocks 1 to 4, and to 1 for the last and fifth block (“Response increase = 1 if random block, else 0”).

#### Prediction Model: Classification of Group (RQ 5)

In an exploratory step, we trained random forests to investigate how accurately the participants’ group (CoI vs HC) could be predicted. The input features were participants’ mean accuracy across blocks, participants’ mean reaction time across the learning phase (refer to RQ 2), age, and participants’ estimated learning curve and response increase. In addition, as features for learning curves (linear and quadratic effect of block) and implicit learning (response increase, coded as explained in the *Implicit Learning (RQ 3) and Learning Curve (RQ 4)* section, we extracted the predicted values of the LME model with the median of the *z*-transformed reaction time per participant per block as the response and learning curve and implicit learning as fixed effects. The model included random slopes for the learning curve and the response increase of the participants.

#### ML and Prediction Model (RQ 5)

ML approaches have shown promising results in predicting potential diagnoses and outcomes. These predictive models combine various parameters that were collected during the study. However, only 1 study by Hong et al [16] used an ML approach to predict participants’ cognitive status. Using a random forest approach [86], they achieved a prediction accuracy of 80.9%. Thus, using ML approaches to predict participants’ potential diagnoses rather than only examining group differences may improve the value of such tasks in cognitive assessments, enabling their broader use in populations of older individuals.

As explained previously, we used the random forest classifier to predict whether a participant belonged to the CoI group or the HC group. Repeated nested leave-one-out cross-validations were used to optimize hyperparameters and gain unbiased estimates of the model performance (eg, [86]). Specifically, each training data set from the initial leave-one-out cross-validation (outer cross-validation) was further split using a subsequent leave-one-out cross-validation (inner cross-validation). In the inner cross-validation, the number of trees per forest (range 10-100 in steps of 10), their maximum depth (range 1-7), and the minimum number of samples in each leaf (range 1-5) were optimized using grid search. Subsequently, to obtain an unbiased measure of accuracy, the best model from the inner cross-validation was used to predict the test set from the corresponding outer cross-validation. Finally, this procedure was repeated 15 times (ie, the same cross-validation procedure with varying random seeds) to account for random variations in the modeling procedure. These ML analyses (RQ 5) were conducted in Julia (version 1.9.3) [87] using the *Machine Learning in Julia* (MLJ, version 0.19.2) library [88].

### Ethical Considerations

The study was approved by the ethics committee of the University Hospital of Tübingen (332/2016BO2). Participation was on a voluntary basis and after written informed consent and signature. Compliance with data protection and the implementation and evaluation were based on relevant regulations, guidelines, and protocols.

## Results

### Response Accuracy (RQ 1)

**Figure 5:**
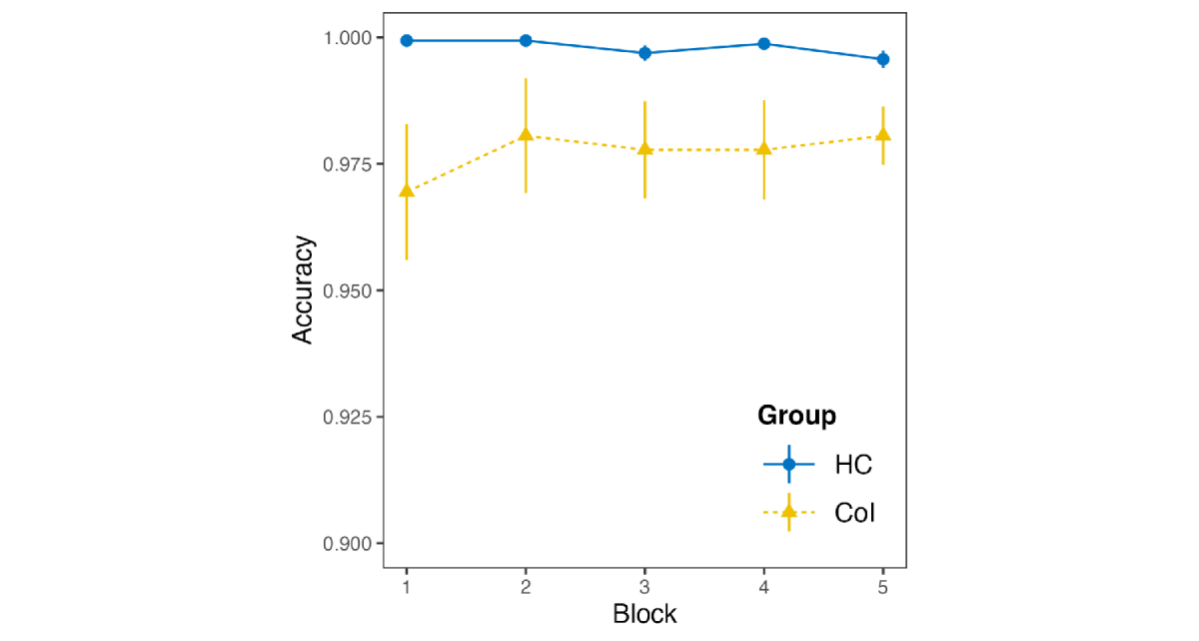
Average response accuracy by group and block. Bars represent the SE of the mean. CoI: participants with cognitive impairment; HC: healthy controls.

The descriptive statistics for the average response accuracy per group are shown in Figure 5. The most suitable generalized linear mixed-effects model using the logistic link function to predict participants’ accuracy was obtained through hierarchical model comparisons, as outlined in the modeling approach in the *General Modeling Approach (RQ 1+RQ 3+RQ 4)* section. The final model contained block, group, and the interaction of block and group as fixed effects and a random intercept for participants. Hierarchical model analyses revealed that age did not contribute significantly to the model fit. The model revealed a significant main effect of group (*b*=−3.64, SE=0.86; *z*=−4.25; *P*<.001). We further found a significant interaction effect between block and group (*b*=0.53, SE=0.23; *z*=2.33; *P*=.02). The main effect of block was not significant (*b*=−0.18, SE=0.11; *z*=−1.54; *P*=.12). The models are listed in Table 2.

**Table 2:** Estimates for participants’ response accuracy for the generalized linear mixed-effects models obtained in the hierarchical modeling approach. The final model is provided in the second column.

Variables	Dependent variable: response accuracy										
	R0: full model containing all fixed and random effects, including interactions^a^				R1: final model, without age as fixed effect^b^				R2: model without age and the interaction between group and block^c^		
	Odds ratio (CI)	*z* value	*P* value	Odds ratio (CI)		*z* value	*P* value	Odds ratio (CI)		*z* value	*P* value
Intercept	270.03 (2.02-36181.50)	2.24	.03	552.97 (238.66-1281.22)		14.73	<.001	332.65 (178.96-618.33)		18.36	<.001
Group	0.02 (0.004-0.15)	−4.02	<.001	0.03 (0.005-0.14)		−4.25	<.001	0.09 (0.03-0.29)		−4.10	<.001
Block	0.84 (0.67-1.05)	−1.54	.12	0.84 (0.67-1.05)		−1.54	.12	1.03 (0.91-1.16)		0.49	.63
Age (y)	1.01 (0.94-1.08)	0.29	.77	—^d^		—^d^	—^d^	—^d^		—^d^	—^d^
Group×block	1.70 (1.09-2.66)	2.33	.02	1.70 (1.09-2.66)		2.33	.02	—^d^		—^d^	—^d^

^a^Observations=225; Bayesian information criterion=420.48.

^b^Observations=225; Bayesian information criterion=415.16.

^c^Observations=225; Bayesian information criterion=416.91.

^d^Variables do not apply to a specific model.

### Average Reaction Times During the Learning Phase (RQ 2)

**Figure 6:**
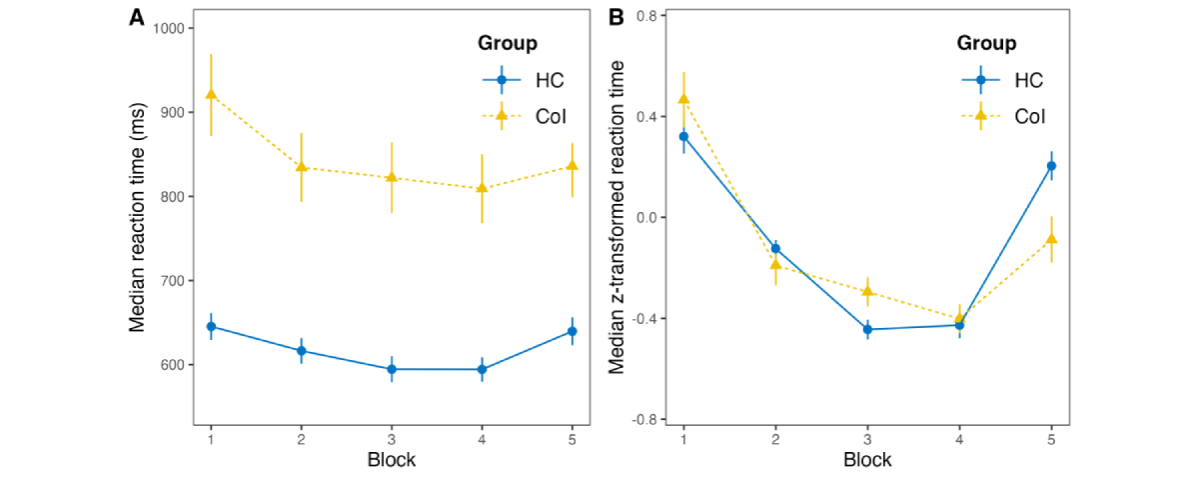
Descriptive statistics of the average reaction times per group and block. Bars represent the SE of the mean. (A) untransformed reaction times and (B) *z*-transformed reaction times. CoI: participants with cognitive impairment; HC: healthy controls.

The ANCOVA comparing participants’ average reaction time (computed as the average of the median reaction times per block) during the learning phase between groups while controlling for age and education revealed a significant difference in the mean reaction times between participants with CoI and HC participants (*F*_1,41_=22.32; *P*<.001), with a large effect size of Cohen *d*_estimated_=1.61 (*η*_p_^2^=0.35). Participants with CoI were, on average, 198.57 (SE 42.03) ms slower than the HCs (during the learning phase). Furthermore, an ANOVA comparing mean reaction times between groups without covariates was conducted to test the robustness of the findings. The results showed a significant difference in reaction time between the participants with CoI and the HC groups (*F*_1,43_=37.02; *P*<.001), indicating a robust effect. Descriptive statistics for the average reaction times during the learning phase are presented in Figure 6, and the results of the statistical analyses are provided in Table 3.

**Table 3:** Estimates for participants’ average reaction time during the learning phase for the analysis of covariance (ANCOVA) and ANOVA models.

Variables	Dependent variable: average reaction time								
	ANCOVA^a^					ANOVA^b^			
	*b* (SE)	*F* test	*η*_p_^2^	*P*	*b* (SE)		*F* test	*η*_p_^2^	*P*
Intercept	526.71 (164.70)	10.23 (1,41)	—^c^	.003	729.52 (19.22)		1440.53 (1,43)	—^c^	<.001
Group	198.57 (42.03)	22.32 (1,41)	0.35	<.001	233.88 (38.44)		37.02 (1,43)	0.46	<.001
Age (y)	3.67 (2.12)	2.99 (1,41)	0.07	.09	—^d^		—^d^	—^d^	—^d^
Education (y)	-4.52 (5.48)	0.68 (1,41)	0.02	.41	—^d^		—^d^	—^d^	—^d^

^a^*R*^2^=0.51, Adjusted *R*^2^=0.47, *F*_3,41_=14.18; *P*<.001.

^b^*R*^2^=0.46, Adjusted *R*^2^=0.45, *F*_1,43_=37.02; *P*<.001.

^c^Not applicable.

^d^Variables do not apply to a specific model.

### Implicit Learning and Learning Curve (RQ 3 and RQ 4)

The descriptive statistics of the average reaction times per group are displayed in Figure 6. As the final model predicting the raw reaction times obtained through the modeling approach outlined in the *Statistical Analysis* section in the *Methods* section resulted in nonnormally distributed residuals, we decided to use *z*-transformation of reaction times over the complete experiment per participant to reduce the effect of baseline differences in reaction times between individuals, which reduced skewness in the distribution of the residuals across participants. That is, we entered the median of the *z*-transformed reaction times per participant per block as the response into the LME. For models predicting the *z*-transformed reaction time, the visual inspection of the residual plot did not suggest a significant deviation from a normal distribution.

The final LME predicting *z*-transformed reaction times contained linear and quadratic terms of block, response increase, group, and the interaction between response increase and block, age, and education as fixed effects and random slopes for linear and quadratic terms of block as well as response increase of participants (formula: *median reaction time [*z*-transformed] ~ time [linear]+ time [quadratic]+response increase+group+age+education in years+response increase: group+time [linear]+ time [quadratic]+response increase|participant*). The hierarchical model analyses revealed that the interaction effect between the linear and quadratic effects of block and group did not significantly improve the model fit. The final model revealed a significant main effect of the linear effect of the block (β=−0.44, SE 0.09; *t*=−4.74; *P*<.001), a significant main effect of the quadratic effect of the block (β=0.46, SE 0.07; *t*=6.59; *P*<.001), a significant main effect of response increase (β=0.23, SE 0.11; *t*=2.21; *P*=.04), and a significant interaction effect of response increase and group (β=−0.34, SE 0.12; *t*=−2.81; *P*=.01). Regarding the interaction effect, participants with CoI had a significantly lower response increase between the random block and the last sequence block compared with HC participants. That is, there was a difference in response increase of *z*-transformed reaction times, obtained from the final model (β=−0.34, SE 0.12), and the difference in untransformed response increase between participants with CoI (mean 26.83, SD 46.09 ms) and HCs (mean 45.37, SD 35.59 ms) was 18.54 ms. The effects of group, age, and education were not significant. The final model, along with the models investigated using the hierarchical modeling approach, is provided in Table 4.

**Table 4:** Estimates for participants’ *z*-transformed response increase of the linear mixed-effects models of the hierarchical modeling approach. The final model is listed in panel C.

Variables		Dependent variable: reaction time (*z*-transformed)		
		*β* (SE)	*t* test	*P* value
**Panel A: R0: full model containing all fixed and random effects, including interactions^a^**				
	Intercept	270.03 (-0.12)	-0.95	.34
	Group	0.07 (0.06)	1.18	.24
	Time (linear)	−0.43 (0.09)	−4.57	<.001
	Time (quadratic)	0.47 (0.07)	6.53	<.001
	Response increase	0.22 (0.11)	1.97	.06
	Age (y)	0.001 (0.002)	0.44	.67
	Education (y)	−0.01 (0.004)	−1.63	.11
	Group × response increase	−0.43 (0.23)	−1.90	.06
	Group × time (linear)	0.02 (0.19)	0.12	.90
	Group × time (quadratic)	0.08 (0.14)	0.57	.57
** Panel B: R1: model without the interaction between group and quadratic time^b^**				
	Intercept	−0.12 (0.12)	−0.97	.34
	Group	0.05 (0.04)	1.08	.29
	Time (linear)	−0.44 (0.09)	−4.65	<.001
	Time (quadratic)	0.46 (0.07)	6.59	<.001
	Response increase	0.23 (0.11)	2.09	.04
	Age (y)	0.001 (0.002)	0.43	.67
	Education (y)	−0.01 (0.004)	−1.63	.11
	Group × response increase	−0.33 (0.15)	−2.24	.03
	Group × time (linear)	−0.03 (0.17)	0.15	.89
** Panel C: R2: final model without the interaction between group and quadratic and linear time^c^**				
	Intercept	−0.12 (0.12)	−0.97	.34
	Group	0.05 (0.04)	1.16	.25
	Time (linear)	−0.44 (0.09)	−4.74	<.001
	Time (quadratic)	0.46 (0.07)	6.59	<.001
	Response increase	0.23 (0.11)	2.09	.04
	Age (y)	0.001 (0.002)	0.43	.67
	Education (y)	−0.01 (0.004)	−1.63	.11
	Group × response increase	−0.34 (0.12)	−2.81	.007
** Panel D: R3: model without the interaction between group and quadratic and linear time, and without the interaction between response increase and group^d^**				
	Intercept	−0.13 (0.12)	−1.04	.30
	Group	−0.03 (0.03)	−1.05	.30
	Time (linear)	−0.44 (0.09)	−4.74	<.001
	Time (quadratic)	0.46 (0.07)	6.59	<.001
	Response increase	0.27 (0.11)	2.33	.02
	Age (y)	0.001 (0.002)	0.44	.66
	Education (y)	−0.01 (0.004)	−1.63	.11

^a^Observations=225; Bayesian information criterion=150.74.

^b^Observations=225; Bayesian information criterion=145.66.

^c^Observations=225; Bayesian information criterion=140.27.

^d^Observations=225; Bayesian information criterion=142.40.

### Prediction Model: Classification of Group (HC vs CoI; RQ 5)

**Figure 7:**
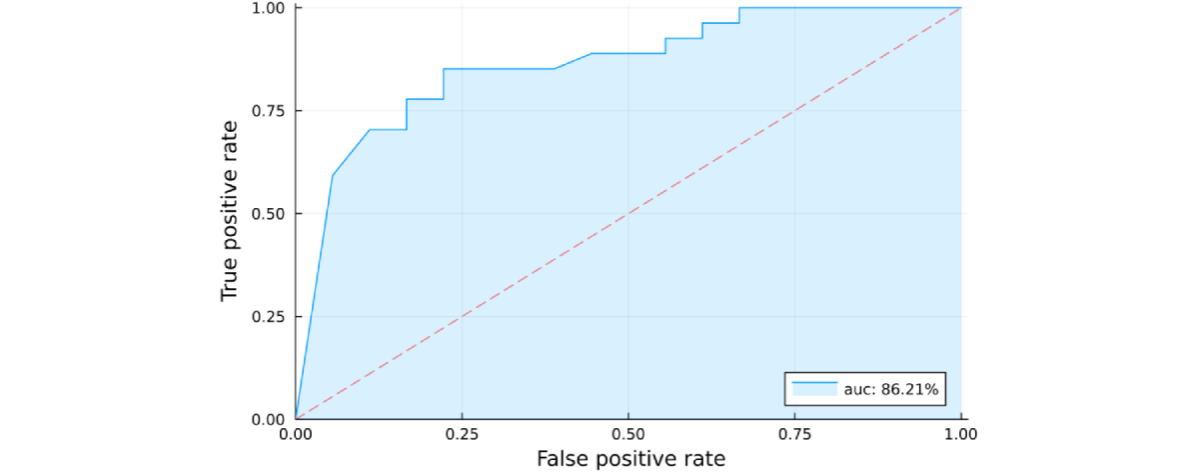
Receiver operating characteristic (ROC) curve for the prediction of cognitive impairment.

Random forest classification predicting the group (CoI or HC) was computed using (1) standardized ordinal linear, (2) quadratic trends in reaction time for the sequence blocks, (3) standardized response increase, (4) age (years), (5) education (years), (6) response accuracy, and (7) average reaction time in milliseconds as features. To extract features 1 to 3, we refitted the LME from RQ 3 without the fixed effects of group, age, and education as well as the corresponding interaction terms. These models showed an average prediction accuracy of 77.13% (95% CI 74.67%-81.33%) across the repeated, nested leave-one-out cross-validation. The receiver operating characteristics curve is shown in Figure 7.

## Discussion

### Principal Findings

#### Overview

In this study, we presented and evaluated a digital tablet–based app featuring a variant of the SRTT to facilitate the diagnosis of implicit learning and memory and use it to predict the assignment of the diagnosis of CoI using an ML modeling approach. The app focuses on use in clinical routines and is based on computer-based studies and the findings of the SRTT. We evaluated our tablet-based SRTT with 27 HC participants and 18 older participants with mild to moderate CoI. We performed statistical analyses to evaluate the replicability and transfer of the results of previous (computer-based) SRTT studies with older participants with CoI to our tablet-based version of the SRTT. In addition, we deployed an ML modeling approach using a random forest classification to predict the participants’ group assignments (HC vs CoI). On the basis of the RQs outlined in the *Introduction* section, the results indicate that we were able to transfer the findings of previous studies to a tablet-based implementation of the SRTT in this study. We found the same significant performance differences between HC and CoI groups, and our ML modeling approach achieves promising results in predicting participants’ group assignments. In summary, our results indicate that the SRTT paradigm is transferable to (touch-based) tablet devices, and the results obtained with our app are comparable with previously published findings. The RQs and their findings are as follows:

1. Do participants with CoI and HC participants differ significantly in response accuracy? That is, do participants with CoI elicit more errors than HC participants?
  - Participants with CoI conducted, on average, significantly more errors per block than HC participants. We found no interaction between the block and the group.
2. Do participants with CoI and HC participants differ in average reaction times during the learning phase? That is, are participants with CoI systematically slower than HC participants?
  - Participants with CoI showed a significantly slower reaction time—on average, approximately 200 ms slower than HC participants (during the learning phase), with a large effect size of Cohen *d*_estimated_=1.61 (η_p_^2^=0.35).
3. Do participants with CoI and HC participants differ in implicit learning? That is, is the response increase in the random block compared with the learning curve significantly lower?
  - Participants with CoI showed a significantly lower response increase than HC participants on *z*-transformed reaction times.
4. Do participants with CoI show a different learning curve during the learning phase than HC participants?
  - No significant differences were observed between participants with CoI and HC participants in terms of linear, quadratic, or cubic learning curves.
5. Can we reliably predict participants’ groups using an ML prediction model?
  - A random forest classification achieved an average prediction accuracy of 77.13%.

In this study, we used a touchscreen-based version of the SRTT. Thus, our results may differ from those of previous studies using keyboard or button box input because of the change in medium. We found the same effects in our tablet-based version previously found in comparable SRTT setups [8,27]. Thus, changing the medium does not significantly change the pattern of implicit learning in older participants with little prior knowledge of technology. The analysis of the parameters of the SRTT with models predicting participants’ groups allows us to make predictions about cognitive status and diagnoses with a relatively high accuracy. Even if only limited statements about isolated and pure implicit learning are possible [71,89], the app can be applied in daily clinical routines with older participants to collect diagnostic neuropsychological information.

#### Response Accuracy (RQ 1)

We found response accuracy to be lower in participants with CoI, in line with previous findings [8,14]. A low response accuracy may indicate difficulties in understanding and memorizing the task instruction, considering the hypothesis of difficulties with task comprehension [8,71,89]. In this study, we altogether excluded 4 participants from the analysis owing to difficulties with the task or attention and behavior difficulties or discontinuation of the examination. Among them, we excluded 2 participants who failed to complete the experiment, 1 participant because of low response accuracy, and 1 with exceptionally prolonged reaction times. This number is comparable with reported exclusions in other studies [8]. After exclusion, participants from the CoI group still had a percentage of correct trials of approximately 95%, compared with approximately 99% to 100% in the HC group. However, both numbers were still very high. Given the assumption of a strong influence of understanding the task instruction as a foremost parameter, as discussed in a recent review [8], we would suppose a lower response accuracy. Thus, the exclusion we made was in a manner that did not result in a systematic methodological error [8]. In summary, we were able to replicate previously published findings on a computer-based SRTT. At the same time, the results indicate that differences in response accuracy are not caused by task incomprehension or methodological errors, indicating the ecological reliability and applicability of our findings.

#### Reaction Time (RQ 2)

Similarly, we found differences in reaction times between the CoI and HC groups in the learning phase (Cohen *d*_estimated_=1.61; *η_p_*^2^=0.35; participants with CoI were, on average, approximately 200 ms slower). This finding follows most previous studies on reaction times in general [90] and in the SRTT in particular [14,16,91]. This finding can be explained by general RT differences in participants with mild and moderate CoI and AD owing to vigilance, cognitive, and psychomotor impairments [92], for example, caused by degeneration of the locus coeruleus [90,93].

#### Response Increase (RQ 3)

As a third factor, we found a significant difference in the response increase between the groups, represented by the difference between the estimated reaction time of the fitted learning curve and the measured reaction time in the random block. As shown in Figure 6, we observed a response increase for both groups. However, the CoI group showed a significantly lower response increase than the HC group, which again is in line with previous findings [8,14]. A lower response increase indicates a less sustainable learning of the sequence. That is, this indicates more than just motor learning and growing familiarity with the task [71]. The response increase in the classical SRTT paradigm has been used as a valuable and verified measure for (differences in) implicit learning [8,24]. Although more basic research scholars recommend a more complex paradigm, for example, with alternating sequences to differentiate different forms of learning more precisely [71,89], we opted for the straightforward approach of contrasting sequenced blocks with a random block to gain a sensitive and specific measure of skill learning for practical use in everyday clinical practice through a short and easy-to-perform task.

#### Learning Curve (RQ 4)

According to RQ 4, all participants showed improved reaction times across the 4 sequence blocks, indicating learning gains in both groups. These findings align with those of previous studies with comparable paradigms and samples [8,14]. With a more differentiated group division, we may find differences in the slope of the curve, which may indicate a more distinguished learning gain in the HC group. Motor learning and familiarity with such tasks certainly interfere with this finding. To what extent motor learning and familiarity with the task affect the learning curve cannot be differentiated at this point.

#### Prediction Model: Classification of Group (RQ 5)

The prediction model obtained through a random forest classification showed an accuracy of 77.13% in predicting the participants’ group (HC vs CoI) correctly. This performance is comparable with that of the study by Hong et al [16], who achieved an accuracy of 80.9% with a similar but lengthier version of the SRTT containing 4 learning and 4 random blocks of 48 trials each (384 trials total). In contrast, our version consisted of 5 blocks of 60 trials each, for a total of 300 trials. In addition, we achieved our results with a more robust ML approach using repeated nested cross-validation. Taken together, we achieved comparable accuracies using only the relatively short and straightforward SRTT paradigm combined with a robust random forest classification. This fact indicates the acceptable accuracy of the diagnosis classifications, despite only a coarse diagnosis classification. This insight is promising for future practical use.

### Implicit Memory as a Part of Digital Neuropsychological Diagnostics

The development of neuropsychological deficits in explicit memory has been thoroughly researched and described and has become an integral part of dementia diagnostics. The role of implicit memory in the diagnosis and distinction of different subtypes of dementia has been scarcely investigated so far. In neuropsychological diagnostics and dementia research, implicit memory can be seen as an additional important domain in the entire pattern of deficits [94]. The use of digital assessment tools [57] can simplify examinations of implicit memory in clinical practice routines; even if overlaps in diseases exist, different participants show different deficit patterns in the process of neurodegeneration [95,96], partly also because of mixed subtypes of dementia [55,56]. This heterogeneity in neurodegeneration can also be seen as a relevant cause of inconsistent research outcomes [8,14] and, of course, needs further research on specific tasks such as the SRTT. On the basis of further research, differential diagnoses can be simplified using a tool similar to the one described in this study.

As different subtypes and mixed subtypes [55,56] have different progression types, paradigms such as the SRTT used for this study can help in the differential diagnosis of different dementia subtypes. When diagnosing the neuropsychological profiles of mixed dementia subtypes, a deeper and more differentiated examination at the level of explicit and implicit memory may be helpful. Using ML prediction can provide further benefit in differentiating diagnostic information based on future clinical studies that include more detailed and comprehensive diagnostics. Even this methodologically broad approach to group classification and the transdiagnostic and heterogeneous CoI group yielded significant results. Therefore, a more sophisticated approach to discriminate diagnosis groups will provide at least comparable results.

One goal of this study was to develop an assessment tool usable in clinical practice without exposing participants to unnecessary strains because of the length and complexity of the task. In developing a tablet-based tool relying on preceding user tests with older participants, we provided a short and transportable assessment instrument suitable even for older participants with CoI.

### Relevance of This Study

In this study, we investigated a touch-based version of the SRTT in a sample of older participants. No control through hardware devices such as keyboards or response boxes was necessary; participants responded directly to the visual target stimuli with their fingers. The response increase and overall high response accuracy, even in participants with CoI, indicate that the paradigm we used is manageable and appropriate for older participants and that the original button-based paradigm is transferrable to tablets.

In our study, exploring an undifferentiated and roughly divided sample, significant differences between the groups were found. The application of statistical models enables the inclusion of features that exceed mere implicit memory, such as response increase. Therefore, the random forest trained achieved a prediction probability of the diagnosis groups of 77.13%. The accuracy of group prediction in our study is comparable with the accuracy reported by Hong et al [16].

Different definitions and concepts of learning are commonly used, based on different memory models, partly as different subtypes. We adhere to “implicit learning” as an umbrella term, as our task is too unspecific to distinguish more sophisticated terms and to differentiate which parts of the process can be explained through motor learning or sequence learning. Our essential objective was not to develop an experimental paradigm for the laboratory to distinguish forms of learning clearly but to provide a simple screening usable as part of a short battery of tests in clinical practice. Such tests could help to distinguish different diagnostic groups in real-life practice.

### Limitations and Strengths

The study was initially part of a technical feasibility study for tablet use that did not address the conventional quality criteria of a clinical trial but had high ecological validity. Trained professional teams made the diagnoses after an extensive examination. Thus, classification into groups was based on the judgments of trained specialists and confirmed by interdisciplinary teams, as neuropsychological and depression scores were not available for all participants.

Divergent paradigms that can distinguish implicit and motor learning more sensitively are available. A more profound distinction between learning processes is not possible with the paradigm used in this study. We intended not to develop a tool for laboratory purposes but a user-centric tool that is usable in the clinic. Using statistical models, we are not limited to the exact distinction.

Taken together, the specific properties of the task partly explain the results found in our study. A pattern of stimuli alternating between random and sequence trials, for example, ensures the discrimination of explicit and implicit memory [32-34]. We chose a more focused approach without alternating patterns, as the use of statistical models for diagnostic information on implicit memory does not depend solely on accurately differentiated implicit memory processes in the experimental paradigm but on a variety of parameters. We also included parameters such as reaction times and learning gains in the statistical model. The objective of the task is not to map implicit learning as accurately as possible but to collect features that can be used in a statistical model to predict diagnostic information. The sequence length can be seen as a second factor. In this case, the well-proven and original sequence by Nissen and Bullemer [24] was used. Thus, we do not expect variations in the sequence or sequence length used in this study to result in the differences we found.

By contrast, a short tool that is easy to use in daily clinical practice is available to assess implicit memory on a tablet, for example, even at the bedside and not only in the laboratory. Because of the nature and implementation of the task, interruptions and early termination by participants who are stressed are less likely. In addition, participants do not receive negative feedback or feelings in the SRTT compared with explicit memory tasks, where they may experience failure in repeating words, drawing figures, or calculating numbers. By not only including pure reaction times but also response increase and response accuracy in a ML model, reliable predictions regarding diagnoses can be made with relatively little data and within a short time. Comparable results, as in previous studies, can be achieved with our app more quickly and simply.

Overall, a reliable assignment of the diagnoses and high ecological validity are possible with the app’s relatively simple and short execution because of the use of ML algorithms. This assignment is preferable for a clinical setting, where brief assessments are essential. The data show that the short procedure is effective and yields results comparable with those obtained with more extended tests.

### Future Studies

In the future, larger samples are needed to test the ability to discriminate similar conditions with heterogeneous cognitive symptom patterns such as dementia and delirium and different dementia subtypes. As implicit impairments are transdiagnostic, the SRTT and similar tasks have been examined with different samples [25]; however, only a few studies were performed with a touchscreen. Shortened versions of the task may facilitate the execution of the task to prevent cognitive overload in participants considered more impaired.

On the basis of more differentiated neuropsychological assessments, the parameters influencing test performance can be identified, especially when implementing additional information into statistical models. The SRTT can also be combined with another short task as another promising way to improve the accuracy of dementia diagnostics with the tablet. For example, this task could be a verbal task addressing different cognitive domains or a proven method such as the clock drawing test.

## Appendix

Multimedia Appendix 1 Pseudorandomized sequence used in block 5.

## Abbreviations

AD: Alzheimer disease
ANCOVA: analysis of covariance
CoI: participants with cognitive impairment
HC: healthy control
LME: linear mixed effect
MCI: mild cognitive impairment
ML: machine learning
RQ: research question
SRTT: serial reaction time task
