# Supplementary material for: Performance Differences of a Touch-Based Serial Reaction Time Task in Healthy Older Participants and Older Participants With Cognitive Impairment on a Tablet: Experimental Study
Source: JMIR Aging. 2024 Mar 21;7:e48265. doi: 10.2196/48265 (PMC10995790; doi:10.2196/48265)
Supplement: Multimedia Appendix 1 [file aging_v7i1e48265_app1.docx]

## Appendix

3131321424 3143213132 4243243231 4214321324 2421321323 2142323243

**Figure 8**: pseudo-randomized sequence used in block 5
